# {6-[2,5-Bis(chloro­meth­yl)-3,4-dihydroxy­tetra­hydro­furan-2-yl­oxy]-3-chloro-4,5-dihydr­oxy-3,4,5,6-tetra­hydro-2*H*-pyran-2-yl}methyl acetate dihydrate

**DOI:** 10.1107/S1600536809053173

**Published:** 2009-12-16

**Authors:** Jing-Yu Zhang, Xue-Hui Hou, Xue-Fen Wu

**Affiliations:** a School of Pharmacy, Henan University of Traditional Chinese Medicine, Zhengzhou 450008, People’s Republic of China; b Department of Humanities and Basic Sciences, Zhengzhou College of Animal Husbandry Engineering, Zhengzhou 450011, People’s Republic of China

## Abstract

The title compound, C_14_H_21_Cl_3_O_9_·2H_2_O, is a disaccharide constructed from a galactose and a fructose. In the mol­ecular structure, the tetra­hydro­furan five-membered ring and tetra­hydro­pyran six-membered ring assume envelope and chair conformations, respectively. An extensive O—H⋯O hydrogen-bonding network occurs in the crystal structure.

## Related literature

For the biological importance of sucrose and its derivatives, see: Liu *et al.* (2004 ▶); Stutz (1999 ▶). 
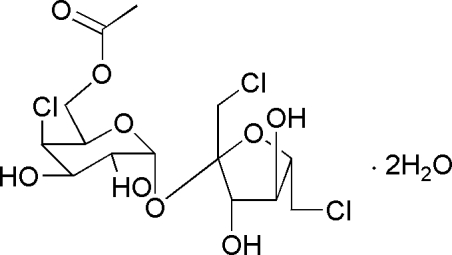


## Experimental

### 

#### Crystal data

C_14_H_21_Cl_3_O_9_·2H_2_O
*M* *_r_* = 475.69
Orthorhombic, 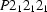

*a* = 7.5824 (8) Å
*b* = 14.2703 (14) Å
*c* = 19.507 (2) Å
*V* = 2110.7 (4) Å^3^
*Z* = 4
Mo *K*α radiation
μ = 0.49 mm^−1^
*T* = 298 K
0.42 × 0.22 × 0.15 mm

#### Data collection

Bruker SMART CCD area-detector diffractometer
Absorption correction: multi-scan (*SADABS*; Sheldrick, 1996 ▶) *T*_min_ = 0.822, *T*_max_ = 0.931
8741 measured reflections
3705 independent reflections
2973 reflections with *I* > 2σ(*I*)
*R*_int_ = 0.042

#### Refinement

*R*[*F* ^2^ > 2σ(*F* ^2^)] = 0.036
*wR*(*F* ^2^) = 0.081
*S* = 1.03
3705 reflections
253 parameters
H-atom parameters constrained
Δρ_max_ = 0.23 e Å^−3^
Δρ_min_ = −0.19 e Å^−3^
Absolute structure: Flack (1983 ▶), 1569 Friedel pairs
Flack parameter: 0.10 (6)

### 

Data collection: *SMART* (Siemens, 1996 ▶); cell refinement: *SAINT* (Siemens, 1996 ▶); data reduction: *SAINT*; program(s) used to solve structure: *SHELXTL* (Sheldrick, 2008 ▶); program(s) used to refine structure: *SHELXTL*; molecular graphics: *SHELXTL*; software used to prepare material for publication: *SHELXTL*.

## Supplementary Material

Crystal structure: contains datablocks I, global. DOI: 10.1107/S1600536809053173/xu2685sup1.cif

Structure factors: contains datablocks I. DOI: 10.1107/S1600536809053173/xu2685Isup2.hkl

Additional supplementary materials:  crystallographic information; 3D view; checkCIF report

## Figures and Tables

**Table 1:** Hydrogen-bond geometry (Å, °)

*D*—H⋯*A*	*D*—H	H⋯*A*	*D*⋯*A*	*D*—H⋯*A*
O3—H3⋯O10	0.82	1.94	2.716 (3)	158
O4—H4⋯O7^i^	0.82	1.88	2.692 (3)	172
O7—H7⋯O3^ii^	0.82	1.81	2.610 (3)	165
O8—H8⋯O11	0.82	2.08	2.844 (3)	156
O10—H10*C*⋯O4^iii^	0.85	1.98	2.820 (3)	171
O10—H10*D*⋯O11^iv^	0.85	2.13	2.972 (3)	171
O11—H11*E*⋯O6^ii^	0.85	2.16	3.011 (3)	176
O11—H11*F*⋯O9^v^	0.85	2.05	2.896 (3)	176

Symmetry codes: (i) 
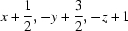
; (ii) 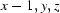; (iii) 
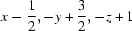
; (iv) 
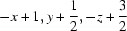
; (v) 
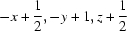
.

**Fig. 1.**
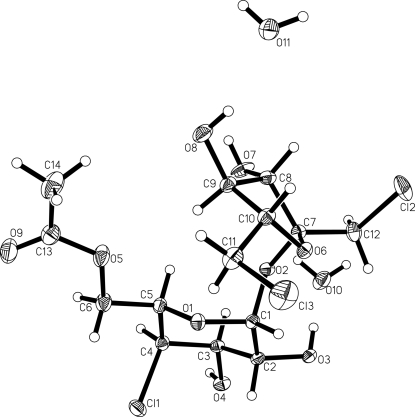
The molecular structure of the compound, with atom labels and 50% probability displacement ellipsoids.

## supplementary crystallographic information

### Comment

Due to its widespread existence in all photosynthetic plants and its biological importance, sucrose and its derivatives are of interest as potentially useful substrates in the chemical and biological fields (Liu *et al.*, 2004; Stutz, 1999). To develop new applications for sucrose and its derivatives, structural modifications of sucrose have been extensively investigated. As a contribution to the sucrose chemistry, we report here the crystal structure of the title compound.

The molecular structure of title compound is shown in Fig.1. Intermolecular hydrogen bonds link molecules in crystal structure into a three-dimensional structure (Table 1).

### Experimental

The reaction was carried out under nitrogen atmosphere. Sucrose (0.50 mol) and thionyl chloride (2.00 mol) were added to a stirred solution of pyridine (500 ml) and stirred at 418 K for 12 h. The solvent was evaporated under vacuum. 50 ml of water was added to the reside and pH was adjusted to 7 with the saturated NaOH-solution. The mixture was washed with toluene (2*30 ml) and concentrated under vacuum to obtain the title compound as a white solid. Colourless crystals suitable for X-ray analysis were obtained by slow evaporation of a ethyl acetate solution over a period of two weeks.

### Refinement

H atoms were positioned geometrically with O—H = 0.82 (hydroxy), 0.85 (water) and C—H = 0.96 (methyl), 0.97 (methylene) and 0.98 Å (methine), and constrained to ride on their parent atoms with *U*_iso_(H) = *xU*_eq_ (C), where *x* = 1.5 for methyl and hydroxyl H atoms and *x* = 1.2 for the others.

### Crystal data

**Table:**

C_14_H_21_Cl_3_O_9_·2H_2_O	*F*(000) = 992
*M**_r_* = 475.69	*D*_x_ = 1.497 Mg m^−^^3^
Orthorhombic, *P*2_1_2_1_2_1_	Mo *K*α radiation, λ = 0.71073 Å
Hall symbol: P 2ac 2ab	Cell parameters from 3263 reflections
*a* = 7.5824 (8) Å	θ = 2.5–25.7°
*b* = 14.2703 (14) Å	µ = 0.49 mm^−^^1^
*c* = 19.507 (2) Å	*T* = 298 K
*V* = 2110.7 (4) Å^3^	Block, colorless
*Z* = 4	0.42 × 0.22 × 0.15 mm

### Data collection

**Table:**

Bruker SMART CCD area-detector diffractometer	3705 independent reflections
Radiation source: fine-focus sealed tube	2973 reflections with *I* > 2σ(*I*)
graphite	*R*_int_ = 0.042
φ and ω scans	θ_max_ = 25.0°, θ_min_ = 1.8°
Absorption correction: multi-scan (*SADABS*; Sheldrick, 1996)	*h* = −8→9
*T*_min_ = 0.822, *T*_max_ = 0.931	*k* = −16→9
8741 measured reflections	*l* = −21→23

### Refinement

**Table:**

Refinement on *F*^2^	Secondary atom site location: difference Fourier map
Least-squares matrix: full	Hydrogen site location: inferred from neighbouring sites
*R*[*F*^2^ > 2σ(*F*^2^)] = 0.036	H-atom parameters constrained
*wR*(*F*^2^) = 0.081	*w* = 1/[σ^2^(*F*_o_^2^) + (0.0283*P*)^2^ + 0.4654*P*] where *P* = (*F*_o_^2^ + 2*F*_c_^2^)/3
*S* = 1.03	(Δ/σ)_max_ = 0.001
3705 reflections	Δρ_max_ = 0.23 e Å^−^^3^
253 parameters	Δρ_min_ = −0.19 e Å^−^^3^
0 restraints	Absolute structure: Flack (1983), 1569 Friedel pairs
Primary atom site location: structure-invariant direct methods	Flack parameter: 0.10 (6)

### Fractional atomic coordinates and isotropic or equivalent isotropic displacement parameters (Å^2^)

**Table:**

	*x*	*y*	*z*	*U*_iso_*/*U*_eq_	
Cl1	1.02954 (11)	0.51019 (6)	0.41375 (4)	0.0512 (2)	
Cl2	0.69677 (15)	0.68921 (6)	0.81804 (4)	0.0696 (3)	
Cl3	0.80356 (16)	0.31574 (7)	0.75058 (6)	0.0844 (4)	
O1	0.8271 (2)	0.49683 (12)	0.55568 (8)	0.0347 (4)	
O2	0.7716 (2)	0.62834 (12)	0.62281 (8)	0.0310 (4)	
O3	1.1081 (2)	0.69493 (13)	0.60293 (9)	0.0364 (5)	
H3	1.0327	0.7349	0.6101	0.055*	
O4	1.1037 (3)	0.70723 (12)	0.45730 (9)	0.0397 (5)	
H4	1.0639	0.7447	0.4295	0.060*	
O5	0.5109 (3)	0.42018 (15)	0.50144 (11)	0.0549 (6)	
O6	0.7424 (2)	0.52803 (11)	0.71868 (9)	0.0329 (4)	
O7	0.4430 (3)	0.67403 (16)	0.62983 (11)	0.0534 (6)	
H7	0.3350	0.6751	0.6278	0.080*	
O8	0.2886 (3)	0.47783 (15)	0.67508 (11)	0.0536 (6)	
H8	0.2590	0.4845	0.7152	0.080*	
O9	0.3805 (4)	0.35928 (18)	0.41001 (13)	0.0728 (8)	
O10	0.9171 (3)	0.85590 (14)	0.60537 (13)	0.0619 (7)	
H10C	0.8167	0.8417	0.5890	0.074*	
H10D	0.9044	0.9015	0.6329	0.074*	
O11	0.0863 (3)	0.51355 (17)	0.79506 (11)	0.0628 (6)	
H11E	−0.0125	0.5198	0.7750	0.075*	
H11F	0.0905	0.5519	0.8283	0.075*	
C1	0.9025 (4)	0.56650 (18)	0.59817 (13)	0.0304 (6)	
H1	0.9602	0.5361	0.6373	0.036*	
C2	1.0379 (4)	0.62448 (18)	0.55956 (13)	0.0299 (6)	
H2	1.1350	0.5825	0.5471	0.036*	
C3	0.9628 (4)	0.66439 (18)	0.49342 (13)	0.0309 (6)	
H3A	0.8761	0.7127	0.5051	0.037*	
C4	0.8711 (4)	0.58870 (19)	0.45181 (13)	0.0339 (7)	
H4A	0.8051	0.6191	0.4148	0.041*	
C5	0.7409 (4)	0.53505 (18)	0.49644 (14)	0.0346 (6)	
H5	0.6492	0.5786	0.5119	0.042*	
C6	0.6536 (4)	0.4552 (2)	0.45939 (16)	0.0471 (8)	
H6A	0.6081	0.4765	0.4156	0.056*	
H6B	0.7385	0.4057	0.4509	0.056*	
C7	0.6951 (4)	0.61492 (17)	0.68933 (13)	0.0295 (6)	
C8	0.4961 (4)	0.61162 (19)	0.68120 (14)	0.0352 (7)	
H8A	0.4398	0.6288	0.7247	0.042*	
C9	0.4633 (4)	0.5094 (2)	0.66658 (14)	0.0369 (7)	
H9	0.4991	0.4967	0.6192	0.044*	
C10	0.5962 (4)	0.46240 (19)	0.71439 (15)	0.0370 (7)	
H10	0.5433	0.4546	0.7599	0.044*	
C11	0.6622 (5)	0.3694 (2)	0.68905 (17)	0.0497 (8)	
H11A	0.7261	0.3782	0.6465	0.060*	
H11B	0.5627	0.3285	0.6799	0.060*	
C12	0.7704 (5)	0.6936 (2)	0.73225 (13)	0.0437 (8)	
H12A	0.7364	0.7532	0.7123	0.052*	
H12B	0.8981	0.6899	0.7315	0.052*	
C13	0.3859 (5)	0.3717 (2)	0.47033 (19)	0.0516 (9)	
C14	0.2524 (5)	0.3352 (3)	0.52023 (19)	0.0766 (12)	
H14A	0.3003	0.2821	0.5441	0.115*	
H14B	0.1479	0.3166	0.4960	0.115*	
H14C	0.2233	0.3834	0.5526	0.115*	

### Atomic displacement parameters (Å^2^)

**Table:**

	*U*^11^	*U*^22^	*U*^33^	*U*^12^	*U*^13^	*U*^23^
Cl1	0.0524 (5)	0.0546 (5)	0.0466 (4)	−0.0006 (4)	0.0119 (4)	−0.0130 (4)
Cl2	0.1087 (9)	0.0690 (5)	0.0311 (4)	0.0230 (6)	−0.0028 (5)	−0.0049 (4)
Cl3	0.0934 (9)	0.0619 (6)	0.0980 (8)	0.0202 (6)	−0.0228 (7)	0.0209 (6)
O1	0.0398 (11)	0.0339 (9)	0.0304 (10)	−0.0023 (9)	−0.0014 (9)	0.0024 (8)
O2	0.0281 (10)	0.0377 (10)	0.0273 (10)	0.0053 (9)	0.0027 (8)	0.0068 (8)
O3	0.0258 (10)	0.0402 (11)	0.0431 (11)	0.0001 (9)	−0.0042 (9)	−0.0064 (9)
O4	0.0352 (11)	0.0427 (11)	0.0413 (11)	−0.0018 (10)	0.0055 (10)	0.0128 (10)
O5	0.0577 (15)	0.0651 (14)	0.0419 (12)	−0.0286 (13)	−0.0010 (12)	−0.0018 (11)
O6	0.0326 (11)	0.0340 (10)	0.0321 (10)	−0.0010 (9)	−0.0045 (9)	0.0071 (8)
O7	0.0249 (12)	0.0672 (15)	0.0679 (15)	0.0043 (11)	−0.0037 (10)	0.0382 (12)
O8	0.0330 (12)	0.0719 (15)	0.0560 (13)	−0.0154 (12)	−0.0016 (10)	0.0056 (12)
O9	0.0735 (19)	0.0873 (18)	0.0577 (16)	−0.0277 (15)	−0.0058 (15)	−0.0145 (15)
O10	0.0496 (15)	0.0453 (13)	0.0908 (18)	0.0043 (11)	−0.0205 (14)	−0.0132 (12)
O11	0.0531 (14)	0.0871 (17)	0.0482 (13)	0.0025 (14)	−0.0063 (11)	−0.0002 (13)
C1	0.0294 (15)	0.0322 (14)	0.0295 (15)	0.0043 (13)	−0.0043 (12)	0.0033 (12)
C2	0.0246 (14)	0.0304 (14)	0.0347 (14)	0.0014 (13)	−0.0021 (12)	−0.0005 (12)
C3	0.0260 (15)	0.0334 (14)	0.0333 (14)	−0.0017 (13)	0.0038 (13)	0.0034 (12)
C4	0.0344 (17)	0.0385 (15)	0.0290 (15)	0.0000 (13)	−0.0031 (13)	0.0020 (12)
C5	0.0333 (16)	0.0392 (15)	0.0314 (14)	−0.0043 (13)	−0.0031 (13)	−0.0014 (12)
C6	0.047 (2)	0.0521 (18)	0.0420 (17)	−0.0170 (16)	0.0015 (16)	−0.0037 (15)
C7	0.0314 (16)	0.0309 (14)	0.0263 (14)	0.0012 (13)	−0.0005 (12)	0.0079 (11)
C8	0.0299 (17)	0.0417 (16)	0.0341 (15)	0.0059 (14)	0.0030 (13)	0.0066 (13)
C9	0.0303 (15)	0.0476 (17)	0.0329 (15)	−0.0062 (15)	0.0003 (12)	0.0065 (13)
C10	0.0321 (16)	0.0425 (17)	0.0362 (16)	−0.0065 (14)	0.0006 (14)	0.0095 (14)
C11	0.052 (2)	0.0373 (17)	0.060 (2)	−0.0049 (16)	−0.0078 (17)	0.0065 (16)
C12	0.057 (2)	0.0391 (16)	0.0346 (16)	−0.0028 (16)	−0.0043 (15)	0.0023 (13)
C13	0.053 (2)	0.0424 (18)	0.059 (2)	−0.0080 (17)	−0.0037 (19)	0.0036 (18)
C14	0.072 (3)	0.084 (3)	0.074 (3)	−0.040 (2)	−0.008 (2)	0.019 (2)

### Geometric parameters (Å, °)

**Table:**

Cl1—C4	1.802 (3)	C2—C3	1.521 (3)
Cl2—C12	1.765 (3)	C2—H2	0.9800
Cl3—C11	1.782 (3)	C3—C4	1.519 (4)
O1—C1	1.415 (3)	C3—H3A	0.9800
O1—C5	1.436 (3)	C4—C5	1.523 (4)
O2—C1	1.412 (3)	C4—H4A	0.9800
O2—C7	1.434 (3)	C5—C6	1.503 (4)
O3—C2	1.418 (3)	C5—H5	0.9800
O3—H3	0.8200	C6—H6A	0.9700
O4—C3	1.418 (3)	C6—H6B	0.9700
O4—H4	0.8200	C7—C12	1.512 (4)
O5—C13	1.321 (4)	C7—C8	1.518 (4)
O5—C6	1.447 (4)	C8—C9	1.507 (4)
O6—C7	1.412 (3)	C8—H8A	0.9800
O6—C10	1.454 (3)	C9—C10	1.528 (4)
O7—C8	1.400 (3)	C9—H9	0.9800
O7—H7	0.8200	C10—C11	1.502 (4)
O8—C9	1.409 (3)	C10—H10	0.9800
O8—H8	0.8200	C11—H11A	0.9700
O9—C13	1.191 (4)	C11—H11B	0.9700
O10—H10C	0.8500	C12—H12A	0.9700
O10—H10D	0.8500	C12—H12B	0.9700
O11—H11E	0.8500	C13—C14	1.498 (5)
O11—H11F	0.8500	C14—H14A	0.9600
C1—C2	1.518 (4)	C14—H14B	0.9600
C1—H1	0.9800	C14—H14C	0.9600
			
C1—O1—C5	112.87 (19)	O6—C7—O2	112.4 (2)
C1—O2—C7	120.57 (18)	O6—C7—C12	109.4 (2)
C2—O3—H3	109.5	O2—C7—C12	104.4 (2)
C3—O4—H4	109.5	O6—C7—C8	105.5 (2)
C13—O5—C6	117.2 (2)	O2—C7—C8	108.2 (2)
C7—O6—C10	110.44 (19)	C12—C7—C8	117.1 (2)
C8—O7—H7	109.5	O7—C8—C9	115.7 (2)
C9—O8—H8	109.5	O7—C8—C7	109.9 (2)
H10C—O10—H10D	108.6	C9—C8—C7	102.3 (2)
H11E—O11—H11F	108.5	O7—C8—H8A	109.5
O2—C1—O1	110.8 (2)	C9—C8—H8A	109.5
O2—C1—C2	107.7 (2)	C7—C8—H8A	109.5
O1—C1—C2	111.4 (2)	O8—C9—C8	116.2 (2)
O2—C1—H1	109.0	O8—C9—C10	114.1 (2)
O1—C1—H1	109.0	C8—C9—C10	101.6 (2)
C2—C1—H1	109.0	O8—C9—H9	108.2
O3—C2—C1	110.1 (2)	C8—C9—H9	108.2
O3—C2—C3	112.4 (2)	C10—C9—H9	108.2
C1—C2—C3	111.8 (2)	O6—C10—C11	109.5 (2)
O3—C2—H2	107.4	O6—C10—C9	104.8 (2)
C1—C2—H2	107.4	C11—C10—C9	114.0 (2)
C3—C2—H2	107.4	O6—C10—H10	109.5
O4—C3—C4	112.7 (2)	C11—C10—H10	109.5
O4—C3—C2	107.5 (2)	C9—C10—H10	109.5
C4—C3—C2	111.0 (2)	C10—C11—Cl3	111.0 (2)
O4—C3—H3A	108.5	C10—C11—H11A	109.4
C4—C3—H3A	108.5	Cl3—C11—H11A	109.4
C2—C3—H3A	108.5	C10—C11—H11B	109.4
C3—C4—C5	110.4 (2)	Cl3—C11—H11B	109.4
C3—C4—Cl1	110.9 (2)	H11A—C11—H11B	108.0
C5—C4—Cl1	110.81 (19)	C7—C12—Cl2	112.3 (2)
C3—C4—H4A	108.2	C7—C12—H12A	109.1
C5—C4—H4A	108.2	Cl2—C12—H12A	109.1
Cl1—C4—H4A	108.2	C7—C12—H12B	109.1
O1—C5—C6	107.4 (2)	Cl2—C12—H12B	109.1
O1—C5—C4	110.8 (2)	H12A—C12—H12B	107.9
C6—C5—C4	113.1 (2)	O9—C13—O5	123.8 (3)
O1—C5—H5	108.5	O9—C13—C14	124.6 (3)
C6—C5—H5	108.5	O5—C13—C14	111.6 (3)
C4—C5—H5	108.5	C13—C14—H14A	109.5
O5—C6—C5	108.6 (2)	C13—C14—H14B	109.5
O5—C6—H6A	110.0	H14A—C14—H14B	109.5
C5—C6—H6A	110.0	C13—C14—H14C	109.5
O5—C6—H6B	110.0	H14A—C14—H14C	109.5
C5—C6—H6B	110.0	H14B—C14—H14C	109.5
H6A—C6—H6B	108.4		
			
C7—O2—C1—O1	96.6 (2)	C10—O6—C7—C8	13.7 (3)
C7—O2—C1—C2	−141.3 (2)	C1—O2—C7—O6	−10.3 (3)
C5—O1—C1—O2	60.7 (3)	C1—O2—C7—C12	108.2 (3)
C5—O1—C1—C2	−59.1 (3)	C1—O2—C7—C8	−126.4 (2)
O2—C1—C2—O3	57.0 (3)	O6—C7—C8—O7	−156.5 (2)
O1—C1—C2—O3	178.7 (2)	O2—C7—C8—O7	−36.0 (3)
O2—C1—C2—C3	−68.7 (3)	C12—C7—C8—O7	81.6 (3)
O1—C1—C2—C3	52.9 (3)	O6—C7—C8—C9	−33.1 (3)
O3—C2—C3—O4	62.5 (3)	O2—C7—C8—C9	87.4 (2)
C1—C2—C3—O4	−173.0 (2)	C12—C7—C8—C9	−155.0 (2)
O3—C2—C3—C4	−173.8 (2)	O7—C8—C9—O8	−77.6 (3)
C1—C2—C3—C4	−49.3 (3)	C7—C8—C9—O8	163.0 (2)
O4—C3—C4—C5	171.3 (2)	O7—C8—C9—C10	158.0 (2)
C2—C3—C4—C5	50.6 (3)	C7—C8—C9—C10	38.6 (3)
O4—C3—C4—Cl1	48.1 (3)	C7—O6—C10—C11	133.7 (2)
C2—C3—C4—Cl1	−72.6 (2)	C7—O6—C10—C9	11.0 (3)
C1—O1—C5—C6	−175.1 (2)	O8—C9—C10—O6	−156.9 (2)
C1—O1—C5—C4	60.9 (3)	C8—C9—C10—O6	−31.1 (3)
C3—C4—C5—O1	−55.8 (3)	O8—C9—C10—C11	83.4 (3)
Cl1—C4—C5—O1	67.5 (2)	C8—C9—C10—C11	−150.8 (2)
C3—C4—C5—C6	−176.5 (2)	O6—C10—C11—Cl3	67.2 (3)
Cl1—C4—C5—C6	−53.2 (3)	C9—C10—C11—Cl3	−175.7 (2)
C13—O5—C6—C5	159.2 (3)	O6—C7—C12—Cl2	−58.1 (3)
O1—C5—C6—O5	67.8 (3)	O2—C7—C12—Cl2	−178.66 (18)
C4—C5—C6—O5	−169.6 (2)	C8—C7—C12—Cl2	61.8 (3)
C10—O6—C7—O2	−104.0 (2)	C6—O5—C13—O9	−3.6 (5)
C10—O6—C7—C12	140.5 (2)	C6—O5—C13—C14	176.7 (3)

### Hydrogen-bond geometry (Å, °)

**Table:**

*D*—H···*A*	*D*—H	H···*A*	*D*···*A*	*D*—H···*A*
O3—H3···O10	0.82	1.94	2.716 (3)	158
O4—H4···O7^i^	0.82	1.88	2.692 (3)	172
O7—H7···O3^ii^	0.82	1.81	2.610 (3)	165
O8—H8···O11	0.82	2.08	2.844 (3)	156
O10—H10C···O4^iii^	0.85	1.98	2.820 (3)	171
O10—H10D···O11^iv^	0.85	2.13	2.972 (3)	171
O11—H11E···O6^ii^	0.85	2.16	3.011 (3)	176
O11—H11F···O9^v^	0.85	2.05	2.896 (3)	176

Symmetry codes: (i) *x*+1/2, −*y*+3/2, −*z*+1; (ii) *x*−1, *y*, *z*; (iii) *x*−1/2, −*y*+3/2, −*z*+1; (iv) −*x*+1, *y*+1/2, −*z*+3/2; (v) −*x*+1/2, −*y*+1, *z*+1/2.

## References

[bb1] Flack, H. D. (1983). *Acta Cryst.* A**39**, 876–881.

[bb2] Liu, F.-W., Liu, H.-M., Yu, K. & Zhang, J.-Y. (2004). *Carbohydr. Res.* **339**, 2651–2656. 10.1016/j.carres.2004.09.013 15519323

[bb3] Sheldrick, G. M. (1996). *SADABS* University of Göttingen, Germany.

[bb4] Sheldrick, G. M. (2008). *Acta Cryst.* A**64**, 112–122. 10.1107/S0108767307043930 18156677

[bb5] Siemens (1996). *SMART* and *SAINT* Siemens Analytical X-ray Instruments Inc., Madison, Wisconsin, USA.

[bb6] Stutz, A. E. (1999). *Iminosugars as Glycosidase Inhibitors: Nojirimycin and Beyond* Weinheim: Wiley–VCH.

